# The neural correlates of emotional conflict monitoring as an early manifestation of affective and cognitive declines in persons with Type 2 diabetes

**DOI:** 10.1093/braincomms/fcad022

**Published:** 2023-02-04

**Authors:** Yu-Hsin Chen, Chenyi Chen, Hong-Yu Jian, Yu-Chun Chen, Yang-Teng Fan, Chih-Yung Yang, Yawei Cheng

**Affiliations:** Department of Physical Medicine & Rehabilitation, National Yang Ming Chiao Tung University Hospital, Yilan 260, Taiwan; Institute of Neuroscience and Brain Research Center, National Yang Ming Chiao Tung University, Taipei 112, Taiwan; Department of Physical Medicine & Rehabilitation, National Yang Ming Chiao Tung University Hospital, Yilan 260, Taiwan; Graduate Institute of Injury Prevention and Control, College of Public Health, Taipei Medical University, Taipei 110, Taiwan; Brain and Consciousness Research Center, Shuang-Ho Hospital, Taipei Medical University, New Taipei City 234, Taiwan; Graduate Institute of Mind, Brain and Consciousness, College of Humanities and Social Sciences, Taipei Medical University, Taipei 110, Taiwan; Psychiatric Research Center, Wan Fang Hospital, Taipei Medical University, Taipei 116, Taiwan; Department of Endocrinology, Taipei City Hospital, Da'an District, Taipei City, 106, Taiwan; Department of Physical Education, National Taiwan University of Sport, Taichung 404, Taiwan; Graduate Institute of Medicine, Yuan Ze University, Taoyuan 320, Taiwan; Department of Education and Research, Taipei City Hospital, Taipei 103, Taiwan; Department of Physical Medicine & Rehabilitation, National Yang Ming Chiao Tung University Hospital, Yilan 260, Taiwan; Department of Education and Research, Taipei City Hospital, Taipei 103, Taiwan; Institute of Neuroscience and Brain Research Center, National Yang Ming Chiao Tung University, Taipei 112, Taiwan

**Keywords:** emotional conflict, Type 2 diabetes, emotional Stroop task, fMRI, MoCA

## Abstract

Patients with Type 2 diabetes are known to be more susceptible to experience dementia and depression/anxiety. The neural circuits of emotional conflict monitoring, as indicated by a Stroop task, might become altered in terms of cognitive and affective impairments in diabetes. This study investigated alterations in the emotional conflict monitoring and associations of corresponding brain activities with metabolic parameters in persons with Type 2 diabetes. Participants with normal cognitive and affective functioning, including 40 persons with Type 2 diabetes and 30 non-diabetes control subjects, underwent a functional MRI paradigm with the face-word emotional Stroop task and detailed cognitive and affective assessments, including the Montreal Cognitive Assessment and Beck Anxiety Inventory. Compared with the controls, people with diabetes exhibited stronger emotional interference, as indicated by differential reaction times between congruent and incongruent trials (Δcon). Δcon was correlated with Montreal Cognitive Assessment test scores and fasting glucose levels. People with diabetes demonstrated altered brain activation and functional connectivity in the neural network for emotional conflict monitoring. The neural network for emotional conflict monitoring mediated the association of pancreatic function with anxiety scores as well as the relationship between Δcon and Montreal Cognitive Assessment scores. Results suggested that alterations in the neural network underlying emotional conflict monitoring might present before clinically measurable cognitive and affective decrements were apparent, thereby bridging the gap between dementia and anxiety/depression in persons with diabetes.

## Teaser

Chen *et al.* report that alterations in the neural network underlying emotional conflict monitoring might already be present before clinically measurable cognitive and affective decrements in Type 2 diabetes were apparent, thereby bridging the gap between dementia and anxiety/depression in diabetes sufferers.

## Graphical Abstract

**Graphical Abstract:**
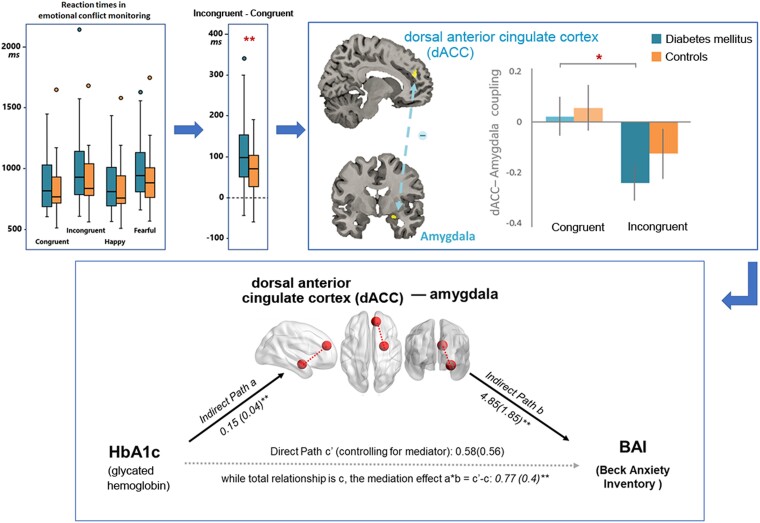

## Introduction

‘Everything about the management makes me feel guilty. Each A1c level is amoral test of how good I can be.’^1^ Once an individual is diagnosed with Type 2 diabetes, apart from managing unwanted symptoms, the psychosocial adjustments with which patient is confronted (e.g. required to stick to a restricted diet, take medications on time and endure a negative body image) are often emotionally stressful.^2^ In fact, the prevalence rate of depression and anxiety in Type 2 diabetes is nearly twice than that of the general population.^3^ It is perspicuous that the psychological burden that patients carry also affects their glycaemic control and quality of life.^4^ Preventive measures and early detection of diabetes-related affective declines are urgently needed in the diabetes community.^5^

Neuroimaging techniques have proven to be useful in identifying biomarkers of mental disorders, even before a condition is clinically diagnosable. In regard to diabetes, most of the magnetic resonance imaging (MRI) literature focused on functional changes related to cognitive impairments and structural abnormalities.^6^ As assessed via blood oxygen level-dependent signals in functional MRI (fMRI), persons with Type 2 diabetes have decreased neural activities in regions implicated in cognitive processing compared to those without the disease.^7^ From structural studies, global brain atrophy in people with Type 2 diabetes is well-documented, with the prefrontal cortices and hippocampus being particularly vulnerable.^8^ However, neuroimaging research that addresses Type 2 diabetes from an affective viewpoint remains elusive.

The cognitive behavioural model suggests that disturbed emotional states due to diabetes are derived from a vicious cycle: low mood and negative beliefs caused by excessive stressors impact diabetes management, which may lead to a poor physical condition and in turn haunts a patient’s psychological status.^2^ The fundamental dysfunction can be attributed to altered emotional processing associated with decrements in cognitive control.^9^ These deficits, commonly presented as affective disorders, are often investigated through the emotional Stroop task,^10^ a paradigm that can be promising for inspecting affective function in persons with diabetes.

Resembling the classic Stroop task, the emotional Stroop task involves an emotional conflict or agreement between task-relevant information and a distractor. When interference occurs, response errors and latencies increase,^10^ with those suffering depression and anxiety disorder sustaining even greater interference effects than normal subjects.^11^ The task comes in two variations: colour-word and face-word emotional Stroop. Some have argued that the former version lacks a consistent behavioural effect since the emotional conflict does not arise from semantic incompatibility.^12^ As a modification, Etkin *et al.*^13^ used facial expressions as targets with words describing emotions printed across it as a distractor. Resolving such emotional conflict engages neural activity in the anterior cingulate cortex (ACC) and lateral prefrontal cortex.^13,14^ At the core of regulating conflicting processes and negative affects,^15^ the ACC is implicated in psychopathology and is of great importance to the current study. Previous studies reported aberrant activation of the ACC in depressed individuals during the emotional Stroop task,^16^ but it is unknown whether these brain activities are altered in Type 2 diabetes.

To assess brain functions of Type 2 diabetes in an affective context, this study sought to examine how people with diabetes might differently respond to emotional conflicts. Applying the face-word emotional Stroop task, we reasonably hypothesized that patients would experience a stronger interference effect and present irregularities in brain activations in regions responsible for emotional conflict monitoring. We thereby aimed to bridge the gap between Type 2 diabetes and diabetes-related affective disorders and provide applicable directions for future studies as well as effective diabetes care.

## Materials and methods

### Participants

Forty-eight people with Type 2 diabetes and 30 control subjects were initially enrolled. In this study, all participants were selected according to the following criteria: (i) middle-age adults, aged 35–64 years; (ii) right-handed; (iii) normal or corrected-to-normal vision; (iv) ≥6 years of education^17,18^ and no probable dementia [with a Montreal Cognitive Assessment (MoCA) score of ≥18]; (v) no current or a history of neurological and psychiatric disorders; (vi) no history of frequent hypoglycaemia; and (vii) no contraindications to imaging procedures. Patients were from the Endocrinology Department of Taipei City Hospital Renai Branch (Taipei, Taiwan), and the diagnosis was confirmed by physicians based on the International Diabetes Federation criteria.^19^ An a priori statistical power analysis (GPower 3.1.9.2 software) was performed using the effect sizes reported in a Type 2 diabetes sample as parameters for our sample size estimation.^20^ Based on previous literature, a final sample of approximately 30 for each group was determined. Eight patients were later excluded due to an inability to complete the experiment (*n* = 5) and excessive head movement during the fMRI scan (>3 mm shift or >3° rotation; *n* = 3), leaving a total of 40 people with Type 2 diabetes and 30 controls eligible for data analyses. All of the participants provided written informed consent for the study, which was approved by the Ethics Committee of National Yang Ming Chiao Tung University and conducted in accordance with the Declaration of Helsinki.

### General procedures

Each participant underwent neuropsychological assessments prior to the fMRI scan. For cognitive functions, the global cognitive ability and working memory were respectively evaluated by the MoCA and Digit Span Test of the Wechsler Memory Scale.^21,22^ The Beck Depression Inventory (1996 revised edition) and the Beck Anxiety Inventory were used to measure depression and anxiety, respectively.^23,24^ An additional Diabetes Distress Scale was administered to patients in order to examine their context-specific distress after being diagnosed with diabetes.^25^ Detailed glycaemic indices were collected from patients through a blood sample in 2 weeks’ time after the experiment, whereas control subjects were requested to provide a report of a recent health examination.

### Experimental paradigm

The face-word emotional Stroop paradigm consists of eight ‘on’ blocks with four congruent (CON) and incongruent (INCON) blocks (14 trials per block, 17.1 ± 4.4 s averaged for all participants) intermixed with eight ‘off’ blocks (15.4 each). In every trial, a word that described a happy or fearful emotion was printed in a bold red font across 1 of 20 facial expressions selected from the Pictures of Facial Affect.^26^ CON and INCON trials were categorized by whether the word and the emotional face in the background were sensibly matched. Participants were instructed to classify the emotional type of the faces as happy or fearful as swiftly and precisely as possible while ignoring the task-irrelevant word superimposed on it. Sequences of the emotional type of the stimuli were pseudo-randomized within each block. Stimuli were executed via MATLAB software (MathWorks, Inc., Sherborn, MA) and were presented by a visual projector in the scanner. The MATLAB codes generated for the experimental paradigm were documented in the Supplementary material. To ensure that participants understood the instructions, each participant completed a practice session (10 trials) of the task before entering the MRI. fMRI and structural MRI data were acquired on a 3-T MRI scanner (Siemens Magnetom Tim Trio, Erlangen, Germany) equipped with a high-resolution 32-channel head array coil (see Supplementary material for MRI acquisition details).

### Task performance analysis

Accuracies and reaction times (RTs) were collected as behavioural measures during fMRI scanning. Extreme responses with an RT faster than 300 ms or slower than 4000 ms were omitted (<0.5% of total data). Moreover, error trials were excluded from the RT analysis. The level of emotional interference, Δcon, calculated by subtracting RTs of CON trials from INCON trials (INCON–CON), was derived as an index to measure response delays caused by the Stroop effect in an emotional setting.^27^ A three-way (two groups × two congruencies × two emotion types) analysis of variance (ANOVA) was applied, while two- sample *t*-tests were also performed across groups to explore simple effects. *P* < 0.05 was accepted as statistically significant.

### Image preprocessing

Image processing and analysis were performed utilizing SPM12 (Wellcome Department of Imaging Neuroscience, London, UK; https://www.fil.ion.ucl.ac.uk/spm) in MATLAB 2020a (MathWorks, Sherborn, MA, USA). For details, please see Supplementary materials.

### Task activation analysis

A two-level approach for block-design fMRI data was adopted. A voxel-by-voxel generalized linear model of expected signal changes for each of the two block categories (CON versus INCON), constructed by convolving the canonical haemodynamic response function, was applied to the smoothed images for each participant. Boxcar regressors, which represented the occurrence of the task conditions, were used to model condition effects at the subject level. Movement parameters were included as nuisance regressors. A high-pass filter (with 128-s cut-off) was applied, and temporal autocorrelations were estimated as an autoregressive model(1). The resulting first-level contrast images were then entered into either a one-sample *t*-test for within-group activation or a two-sample *t*-test for between-group comparisons. To determine if brain activity was correlated with task performance, group-level covariate analyses adding Δcon as a covariates were computed. Whole-brain activations were corrected for multiple comparisons with a family-wise error rate at *P* < 0.05. Monte Carlo simulation implemented using 3dClustSim (https://afni.nimh.nih.gov/pub/dist/doc/program_help/3dClustSim.html) determined that a 20-voxel extent at a height threshold of *P* < 0.005 uncorrected yielded a family-wise error-corrected threshold of *P* < 0.05, accounting for spatial correlations in neighbouring voxels (significance level: voxel *P* = 0.005, *α* = 0.05 with 5000 Monte Carlo simulations after grey matter masking). Activities of regions of interest were extracted using the MarsBaR toolbox (http://marsbar.sourceforge.net/) installed in SPM12. Regions of interest for the dACC (4, 36, 24), dorsolateral prefrontal cortex (DLPFC: 44, 34, 14) and ventrolateral prefrontal cortex (VLPFC: −38, 26, 0) were reported for significant contrast image peaks within 10 mm of a priori coordinates that were determined on the basis of a recent fMRI meta-analytical study that compared neural substrates of emotional Stroop tasks in healthy and clinical populations.^28^

### Functional connectivity analysis

The psychophysiological interaction (PPI) analysis assesses the hypothesis that the activity in one brain region can be explained by an interaction between cognitive processes and haemodynamic activity in another brain region. Based on prior literature,^15^ the current study examined whether coupling between the ACC and amygdala (AMY) exhibited distinct patterns between task congruency. The PPI analysis was seeded in the dACC (*x* 10, *y* 44, *z* 24, obtained from results of the group-level covariate analysis) and was performed for each group and condition (CON and INCON) separately. As the physiological variable, the individual time series of the dACC was acquired by extracting the first eigenvariates of the subject-specific contrast signals in a 4-mm-radius sphere centred at the coordinates of the source region. Task congruency was the psychological regressor, which was used as a vector coding for the specific task convolved with the haemodynamic response function. The product of the physiological and psychological variables constituted the third regressor, the interaction term. PPI analyses were conducted by creating a design matrix with those three regressors, with the interaction term being the regressor of interest. For each subject, it generated a whole-brain contrast image indicating the sign and magnitude of functional couplings for all voxels about the source region. A mask encompassing the bilateral AMY was derived from the Harvard–Oxford cortical and subcortical structural atlases. Obtained by a two-way ANOVA (two groups × two congruencies), group results were masked and thresholded at a statistical criterion of ≥10 contiguous voxels at an uncorrected *P* < 0.005.

### Statistical and correlational analyses

Demographic and neuropsychological data were compared between groups by using a two- sample *t*-test (for continuous variables) or chi-squared test (for categorical variables). To identify whether task performance was associated with neuropsychological measures or glycaemic indices within the diabetes group, correlational analyses were carried out by either a Pearson correlation or a non-parametric Spearman rank correlation. Partial correlations adjusted for gender, age and educational level were applied when clinical variables were involved. Task performance was assayed by the level of emotional interference (Δcon). These analyses were performed with SPSS 24.0 (IBM, Armonk, NY, USA), with statistical significance accepted at *P* < 0.05.

### Mediation analyses

To examine interrelationships among conflicting monitoring system, cognitive and emotional function, and diabetes parameters, Mediation Effect Parametric Mapping was used to test specific hypotheses about brain–behaviour relationships.^29^ Herein, intrigued by our behavioural results, where the level of emotional interference, Δcon, was negatively associated with the global cognitive ability (MoCA) in the group with diabetes but not in the control group and the significant association between glycated haemoglobin levels and Beck Anxiety scores found in the diabetes group, we were curious whether these associations were mediated and predicted by a common neural mechanism emphasizing cognitive control regions for a transdiagnostic understanding of clinical disorders as recently reported in an fMRI meta-analysis study.^28^ In the mediation analysis model, path **a** coded the link in which the predictor variable (Δcon for the Δcon–MoCA model and HbA1c for the HbA1c–anxiety model) had to be related to the mediator. The mediator was the neural mechanism underlying emotional conflict monitoring. Path **b** coded the link in which the mediator had to be directly related to the outcome. The mediation effect (**a**∗**b**) had significance, which amounted to a statistical test on the product of the **a** and **b** path coefficients. Equivalently, the test for the predictor–outcome relationship was significantly reduced by inclusion of the mediator in the path model. We referred to the overall predictor–outcome relationship as the **c** effect and controlled the direct effect for the mediator as **c′**. The **a**∗**b** effect was to test the significance of **c − c'** as stated in another study.^30^

### Data availability

The data that support the findings of this study and the code used for data analysis are available upon reasonable request to the corresponding author. The generated codes for the experimental paradigm are available in the Supplementary material.

## Results

### Participant characteristics

Demographics, neuropsychological assessments results and clinical characteristics of participants are summarized in Table 1. Control subjects were matched with patients in age and educational level but not gender. Although the two groups significantly differed in global cognitive ability (MoCA) and anxiety level (Beck Anxiety Inventory), diabetes patients’ average cognitive and affective statuses [MoCA, Beck Depression Inventory (1996 revised edition), Beck Anxiety Inventory and Diabetes Distress Scale] were all within non-clinical ranges.

**Table 1:** Demographic, neuropsychological and clinical characteristics of participants.

	Group			
	Unit	DM	Control	*P*
Age	years	51.4 (6.5)	48.7 (8.8)	0.167
BMI	kg/m2	27.2 (5.2)^a^	23.0 (2.2)	< 0.001***
FPG	mg/dl	142.6 (33.5)^a^	87.7(9.6)^a^	< 0.001***
Educational attainment	years	14.7 (2.5)	15.7 (2.5)	0.096
*N* (female)^b^		40 (10)	30 (20)	< 0.001***
MoCA		27.7 (2.1)^a^	29.5 (0.6)^a^	< 0.001***
DST		23.2 (3.9)	23.7 (4.7)	0.586
BDI-II		7.8 (6.9)^a^	6.3 (7.2)^a^	0.360
BAI		6.9 (7.1)^a^	3.5 (3.0)^a^	0.009**
DDS		37.5 (12.0)	N/A	
HOMA2-IR		2.5 (1.7)^a^	N/A	
HbA1c	%	7.4 (1.5)^a^	N/A	
Postprandial glucose	mg/dl	190.0 (58.7)	N/A	
Fasting insulin	µIU/ml	15.5 (17.4)^a^	N/A	
Postprandial Insulin	µIU/ml	51.7 (45.0)^a^	N/A	
Fasting C-peptide	ng/ml	3.0 (1.9)^a^	N/A	
Postprandial C-peptide	ng/ml	6.7 (3.9)	N/A	
SBP	mmHg	127.0 (7.7)	N/A	
DBP	mmHg	79.9 (6.7)	N/A	
TG	mg/dl	157.7 (107.3)	N/A	
LDL-C	mg/dl	100.1 (24.9)	N/A	
Duration of DM	years	7.8 (3.8)	N/A	

DM, diabetes mellitus; BMI, body mass index; FPG, fasting plasma glucose; MoCA, Montreal Cognitive Assessment; DST, Digit Span Test; BDI-II, Beck Depression Inventory-II; BAI, Beck Anxiety Inventory; DDS, Diabetes Distress Scale; HOMA2-IR, updated homoeostatic model assessment–insulin resistance; HbA1c, glycated haemoglobin; SBP, systolic blood pressure; DBP, diastolic blood pressure; TG, triglyceride; LDL-C, low-density lipoproteins cholesterol. a data that were not normally distributed (Shapiro–Wilk test, *P >* 0.05); b chi-squared test. ** *P* < 0.01; *** P < 0.001.

### Emotional Stroop performance

**Figure 1:**
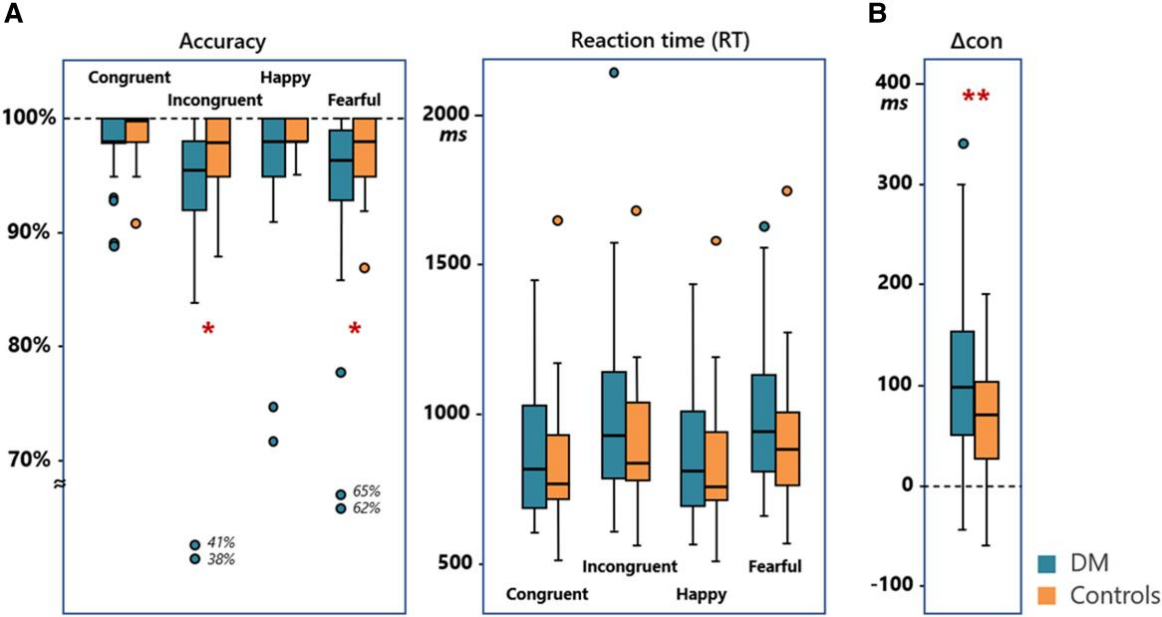
**Behavioural performance on the emotional Stroop task between the diabetes and control groups.** (**A**) A three-way ANOVA (group, congruency and emotion type) for accuracy yielded significant main effects of the factor group (*F*_1,68_ = 4.69, *P* = 0.034, *pη*^2^ = 0.065), congruency (*F*_1,68_ = 10.19, *P* = 0.002, *pη*^2^ = 0.13) and emotion type (*F*_1,68_ = 22.90, *P* < 0.001, *pη*^2^ = 0.252). While the accuracy was lower for fearful faces (fearful versus happy: 95.3% ± 0.8% versus 97.5% ± 0.6%) and for incongruent conditions (incongruent versus congruent: 94.5% ± 1.2% versus 98.3% ± 0.3%), persons with Type 2 diabetes were more error-prone than were the controls (diabetes versus controls: 95% ± 0.9% versus 97.8% ± 1%). (**B**) In terms of emotional interference (Δcon) and differences in the relative reaction times between incongruent trials and congruent trials, patients experienced significantly stronger interference effects (114 ± 14 versus 63 ± 11 ms, *t* = 2.781, *P* = 0.007).

A three-way ANOVA (group, congruency and emotion type) for accuracy yielded significant main effects of the factor group (*F*_1,68_ = 4.69, *P* = 0.034, *pη*^2^ = 0.065), congruency (*F*_1,68_ = 10.19, *P* = 0.002, *pη*^2^ = 0.13) and emotion type (*F*_1,68_ = 22.90, *P* < 0.001, *pη*^2^ = 0.252). While the accuracy was lower for fearful faces (fearful versus happy: 95.3% ± 0.8% versus 97.5% ± 0.6%) and for INCON conditions (INCON versus CON: 94.5% ± 1.2% versus 98.3% ± 0.3%), persons with Type 2 diabetes were more error-prone than were the controls (diabetes versus controls: 95% ± 0.9% versus 97.8% ± 1%) (Fig. 1A). Four additional variables [body mass index (BMI), age, gender and educational status] were further included as nuisance covariates in a sensitivity test using ANCOVA model. After controlling for the BMI, age, gender and educational status, the group × congruency interaction (*F*_1,64_ = 3.993, *P* = 0.05, *pη*^2^ = 0.06) was found to be significant. *Post hoc* analysis revealed a larger effect of congruency in patients (CON versus INCON: 98% ± 0.5% versus 91.2% ± 1.8%) relative to controls (98.6% ± 0.5% versus 98% ± 2.1%), leaving patients with stronger emotional interference than controls.

As of RTs, significant main effects of factor congruency (*F*_1,68_ = 39.50, *P* < 0.001, *pη*^2^ = 0.367), emotion type (*F*_1,68_ = 57.16, *P* < 0.001, *pη*^2^ = 0.457) and the group × congruency interaction (*F*_1,68_ = 5.43, *P* = 0.023, *pη*^2^ = 0.074) were observed. *Post hoc* analysis showed that the factor group exerted a larger effect in an INCON (*F*_1,68_ = 2.995, *P* = 0.088, *pη*^2^ = 0.042) relative to a CON condition (*F*_1,68_ = 0.678, *P* = 0.41, *pη*^2^ = 0.01), leaving patients with stronger emotional interference (Δcon: 114 ± 14 versus 63 ± 11 ms; *P* = 0.007) than controls (Fig. 1B). The sensitivity test results showed that, after controlling for the BMI, age, gender and educational status, the group × congruency interaction remained significant (*F*_1,64_ = 5.447, *P* = 0.023, *pη*^2^ = 0.078). *Post hoc* analysis revealed a larger effect of congruency in patients (CON versus INCON: 834.47 ± 36.25 ms versus 979.51 ± 47.69 ms) relative to controls (887.61 ± 43.22 ms versus 940.29 ± 56.88 ms), indicating patients with stronger emotional interference than controls.

The level of emotional interference, Δcon, was negatively associated with the global cognitive ability (MoCA scores) in the diabetes group (*r*_s_ = −0.478, *P* = 0.002), but not in the control group (*r*_s_ = −0.002, *P* = 0.992). There was also a positive correlation between Δcon and fasting glucose levels in patients after correcting for gender, age and education (*r*_s_ = 0.330, *P* = 0.049). Detailed correlational results are given in Supplementary Table 1. After controlling for additional covariates of vascular risks (BMI, systolic blood pressure, diastolic blood pressure, triglyceride and low-density lipoproteins cholesterol) and diabetes disease duration, the Δcon–MoCA correlation remained significant (*r*_s_ = −0.51, *P* < 0.001).

### fMRI activations

**Figure 2:**
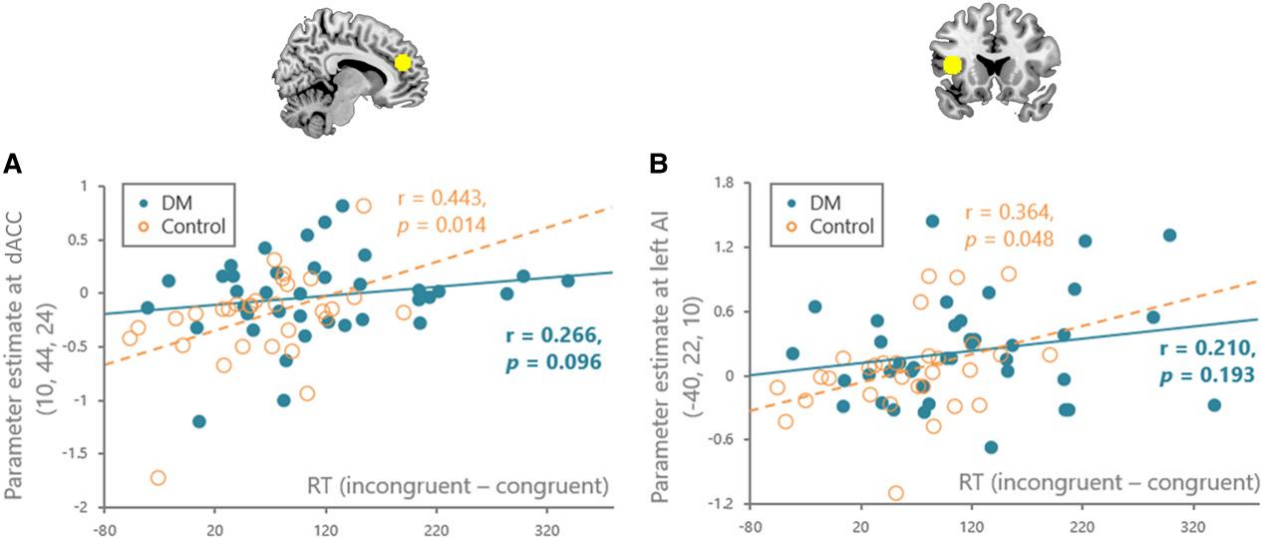
**Associations between emotional interference (Δcon) with the dorsal anterior cingulate cortex (dACC) and anterior insula (AI) activity.** (**A**) The dACC activity was positively correlated with Δcon in the controls (*r* = 0.443, *P* = 0.014) but none in the diabetes (*r* = 0.266, *P* = 0.096). (**B**) The AI activity was positively correlated with Δcon in the controls (*r* = 0.364, *P* = 0.048) but none in the diabetes (*r* = 0.210, *P* = 0.193).

Neural correlates of emotional conflicts (INCON–CON) induced common responses in the lateral prefrontal cortex, medial frontal cortex and ACC in both groups (Supplementary Table 1, Supplementary Fig. 1). Whole-brain between-group comparisons showed decreased activation in the left VLPFC and left hippocampal regions in the diabetes group relative to the controls (Supplementary Table 2, Supplementary Fig. 2). Patients also had greater activity in the ACC, as revealed by a region of interest analysis. After controlling for the additional covariates of BMI, age, gender and educational status, the main effect of group remained significant for ACC (*F*_1,64_ = 4.293, *P* = 0.042, *pη*^2^ = 0.063). Collapsed across groups, the dorsal anterior cingulate cortex (dACC: *x* 10, *y* 44, *z* 26) and anterior insula (*x* −40, *y* 22, *z* 10) emerged as regions where Δcon was correlated with brain responses (Supplementary Table 2). Nonetheless, those associations were only statistically significant in controls (controls: dACC, *r* = 0.443, *P* = 0.014; anterior insula, *r* = 0.364, *P* = 0.048; DM: dACC, *r* = 0.266, *P* = 0.096; anterior insula, *r* = 0.210, *P* = 0.193; Fig. 2). After controlling for the additional covariates of BMI, age, gender and educational status, the correlation patterns remained unchanged for dorsal ACC (controls: *r*_s_ = 0.453, *P* = 0.026; DM: *r*_s_ = 0.231, *P* = 0.187).

### Functional connectivity

**Figure 3:**
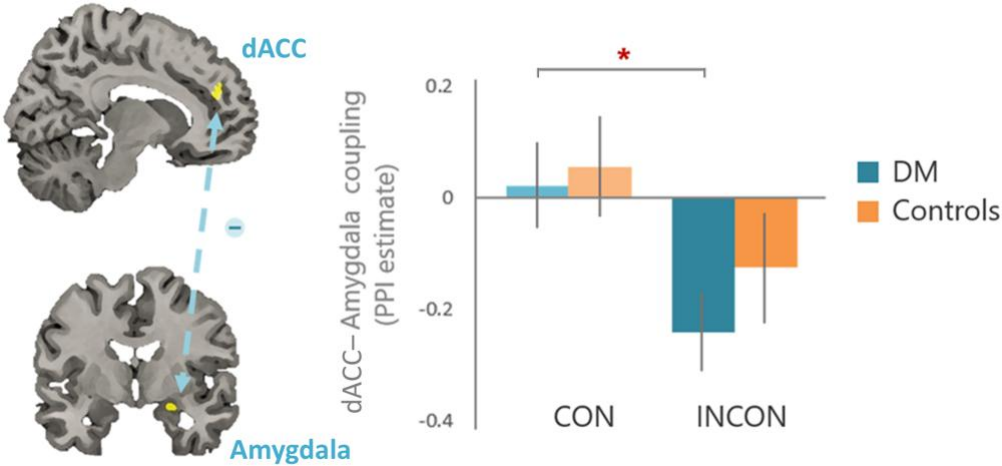
**The functional connectivity between the dorsal anterior cingulate cortex (dACC) and amygdala (dACC–AMY).** The psychophysiological interaction analysis showed a main effect of factor congruency (*F*_1,68_ = 5.91, *P* = 0.018, *pη*^2^ = 0.08) as participants exhibited negative functional coupling between the dACC and amygdala (*x* 26, *y* 4, *z* 16, *k* = 18; dACC–AMY) during incongruent trials [*M*(INCON) = −0.186 (0.059), *M*(CON) = 0.042(0.06), respectively, Bonferroni-corrected *P* < 0.05]. *Pre hoc* planned group-wise comparisons indicated that the dACC–AMY connectivity differed by congruency in diabetes but not in controls, with diabetes showing more-negative dACC–AMY connectivity in incongruent trials than in congruent trials [diabetes: *M*(INCON) = −0.241(0.069), *M*(CON) = 0.02(0.079), *t* = −2.061, *P* = 0.046; controls: *M*(INCON) = −0.13(0.099), *M*(CON) = 0.064(0.091), *t* = −1.439, *P* = 0.161].

The PPI analysis showed a main effect of factor congruency (*F*_1,68_ = 5.91, *P* = 0.018, *pη*^2^ = 0.08) as participants exhibited negative functional coupling between the dACC and AMY (*x* 26, *y* 4, *z* 16, *k* = 18; dACC–AMY) during INCON trials [*M*(INCON) = −0.186 (0.059), *M*(CON) = 0.042(0.06), respectively, Bonferroni-corrected *P* < 0.05]. *Pre hoc* planned group-wise comparisons indicated that the dACC–AMY connectivity differed by congruency in diabetes but not in controls, with diabetes showing more-negative dACC–AMY connectivity in INCON trials than in CON trials [diabetes: *M*(INCON) = −0.241(0.069), *M*(CON) = 0.02(0.079), *t* = −2.061, *P* = 0.046; controls: *M*(INCON) = −0.13(0.099), *M*(CON) = 0.064(0.091), *t* = −1.439, *P* = 0.161], although no group difference (*F*_1,68_ = 1.141, *P* = 0.289, *pη*^2^ = 0.016) or group × congruency interaction (*F*_1,68_ = 0.127, *P* = 0.722, *pη*^2^ = 0.002) reached significance under the omnibus ANOVA test (Fig. 3). After controlling for the additional covariates of BMI, age, gender and educational status, an additional BMI × congruency interaction was identified in people with Type 2 diabetes (*F*_1,35_ = 5.672, *P* = 0.023, *pη*^2^ = 0.139). Patient’s obesity level parametrically modulated the intensity of dACC–AMY connectivity, depending on the effect of congruency, in which BMI positively correlated with the dACC–AMY connectivity during the CON condition (*r*_s_ = 0.314, *P* = 0.048) but negatively correlated with the dACC–AMY connectivity in the INCON condition (*r*_s_ = −0.331, *P* = 0.037).

### Mediation results

**Figure 4:**
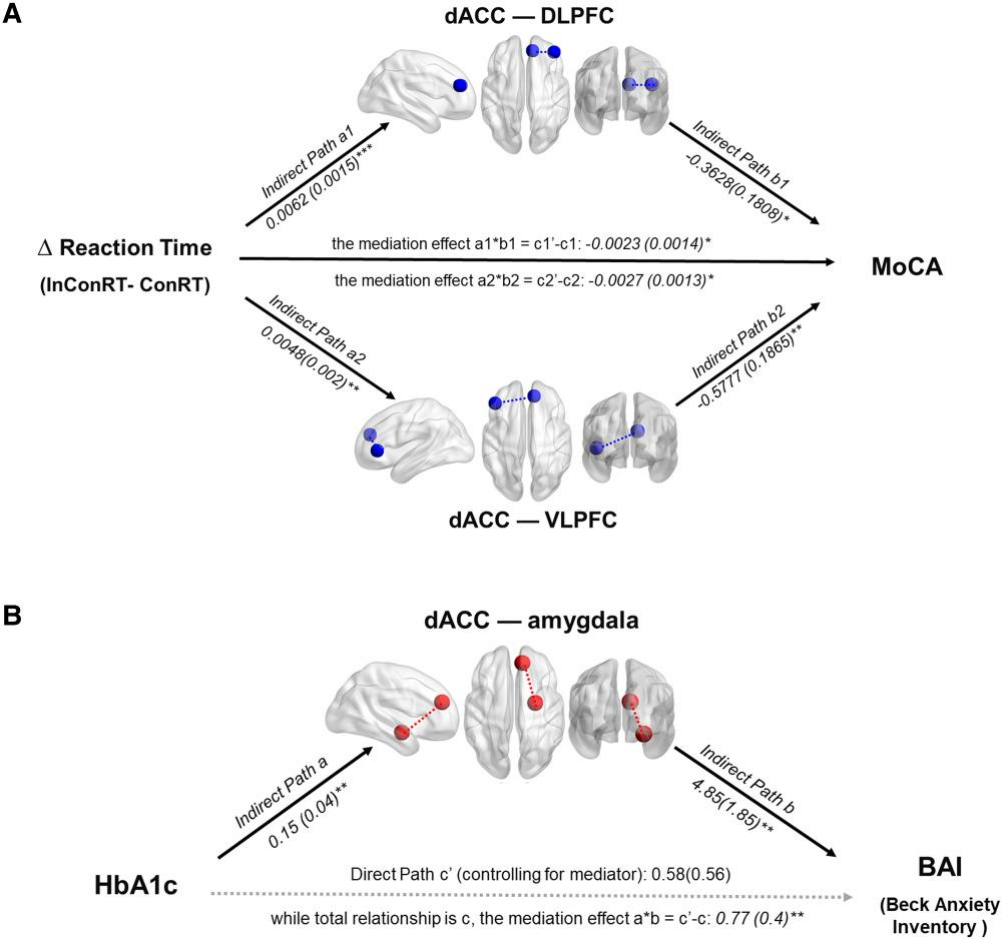
**The functional connectivity mediates glucose levels, behavioural interference, cognitive and affective declines.** (**A**) The dorsal anterior cingulate cortex (dACC)–dorsolateral prefrontal cortex (DLPFC) functional connectivity and dACC–ventrolateral PFC (VLPFC) functional connectivity mediate the effects of behavioural interference (Δcon) on the prediction of Montreal Cognitive Assessment (MoCA) scores in individuals with diabetes. (**B**) The dACC–amygdala functional connectivity mediates the association between glycated haemoglobin (HbA1c) and the prediction of Beck Anxiety scores in individuals with diabetes. Functional connectivity was visualized by the lateral, top and back views of glass brain visualization.

dACC–DLPFC functional connectivity and dACC–VLPFC functional connectivity mediated the effects of behavioural interference (Δcon) on the predicted progression of MoCA cognitive decline in persons with Type 2 diabetes. While a larger Δcon predicted greater cognitive decline, dACC–DLPFC and dACC–VLPFC functional connectivity significantly mediated this Δcon–MoCA association. During INCON emotional conflict monitoring, dACC–DLPFC and dACC–VLPFC functional connectivities were positively associated with Δcon and negatively predicted MoCA scores (dACC–DLPFC: *a*1 = 0.0062, *Z* = 3.9064, *b*1 = −0.3628, *Z* = −2.0767 and *a*1*b1 = −0.0023, *Z* = −1.9856; dACC–VLPFC: *a*2 = 0.0048, *Z* = 2.8572, *b*2 = −0.5777, *Z* = −3.2480 and *a*2*b2 = −0.0027, *Z* = −2.4353, all *P* < 0.05; Fig. 4A; Supplementary Fig. 3; Supplementary Fig. 4). The dACC–AMY functional connectivity mediated the association between HbA1c and Beck Anxiety scores in people with Type 2 diabetes (*a* = 0.1543, *Z* = 2.8771, *b* = 4.8451, *Z* = 2.7617 and a*b = 0.7743, *Z* = 2.6632, all *P* < 0.05; Fig. 4B; Supplementary Fig. 5). While an increased HbA1c level predicted higher anxiety, dACC–AMY functional connectivity significantly mediated this HbA1c–anxiety association. During INCON emotional conflict monitoring, dACC–AMY functional connectivity was positively associated with HbA1c and positively predicted Beck Anxiety scores.

## Discussion

This study used an emotional Stroop fMRI paradigm to evaluate alterations in behaviours and brain patterns in persons with Type 2 diabetes. People with Type 2 diabetes, with relatively normal psychological states, exhibited deficits when encountering emotional conflicts, as evaluated by the face-word emotional Stroop task. Stronger emotional interference effects, decreased prefrontal and hippocampal activation, and increased engagement of the ACC were identified in the patient group compared to their counterparts. Patients with poorer glycaemic control showed more exaggerated emotional Stroop interference and more aberrant brain activations and thus would likely experience greater anxiety.

Biased information processing, especially toward stimuli with a negative valence, was linked to depressive symptomatology and was evinced in meta-analyses studying individuals suffering depression and anxiety disorders.^31^ Our results demonstrated that people with Type 2 diabetes experienced such biases as they responded less accurately relative to a control group. Furthermore, with the implementation of the face-word emotional Stroop task, this study was able to determine the interference effect on the basis of congruency. In line with our expectations, we found that persons with Type 2 diabetes sustained significantly stronger emotional interference effects than normal subjects. Instead of refocusing on appropriate emotional information, patients had difficulty overriding prepotent responses in conflicting scenarios, implying that Type 2 diabetes caused impairments of inhibitory control coupled with emotional processing.^9^ Past studies indicated that poor cognitive control is a precursor to developing depression and ruminative thinking.^32^ Conversely, subjects with a better performance on the emotional Stroop task were less susceptible to stress in daily life that led to finer emotional regulation.^33^

At the brain level, people with Type 2 diabetes decreased the VLPFC and hippocampus activation when experiencing emotional conflicts as compared to the control group. Atrophy and reduced functional activation were widely reported in the prefrontal cortex and hippocampus of the diabetes.^7,8^ Both of these regions are specific areas that are insulin-sensitive in the human brain^34^; hence, behaviours associated with those regions could be altered in conditions where insulin and glucose levels are dysregulated.^35^ The VLPFC was implicated in inhibitory control of emotional distractions. The more greatly the VLPFC is activated, the more efforts are engaged to complete an ongoing task and bring down the influence of undesired emotions.^36^ Comparably, one study employing the face-word emotional Stroop paradigm discovered hypoactivation of the left VLPFC in subjects with major depressive disorder,^37^ indicating that functional irregularities regarding emotional conflict processing may be shared between Type 2 diabetes and depression. The hippocampus, which is considered the earliest affected region in Type 2 diabetes,^38^ is also involved in response to conflicts.^39^

Herein, the pattern of dACC-centred activation and functional connectivity appeared distinct in Type 2 diabetes. ACC activation was significantly greater in patients than controls in response to emotional conflicts. Moreover, in analysing the PPI, negative functional connectivity between the dACC and AMY is critical in INCON situations. Representing top-down control, negative dACC–AMY functional couplings were indicated to be key to emotional conflict resolution and effective emotional regulation.^13,15,40^ Although the group variable did not yield significance in dACC–AMY connectivity, the preplanned group-wise comparison yielded that people with Type 2 diabetes showed exceptionally heightened dACC–AMY connectivity during INCON conflict monitoring. In line with current findings that there is abundant evidence showing exaggerated emotional Stroop interference at both the behavioural and neural levels across a variety of patient populations,^28^ the increased dACC activation and dACC–AMY connectivity, together with the larger psycho-behavioural interference, Δcon, found in the diabetes group suggest a less efficient modulating role of this aforementioned circuit, and it could serve as a transdiagnostic tool to predict cognitive declination in people with diabetes. The inefficient engagement of the conflict resolution system in Type 2 diabetes was further evinced by the smaller Pearson correlation coefficient between Δcon and dACC activity and by that between Δcon and left anterior insula activity in the patient group than the control group. The stronger but less efficient engagement was supported by a recent study also showing higher resting state functional connectivity in Type 2 diabetes.^41^ Although the MoCA and anxiety scores of most of our diabetes group did not meet the clinical cut points (MoCA: 23; Beck Anxiety Inventory: 16),^24^ early symptoms of cognitive declination were predicted by our machine learning model using neuropsychological profile of cognitive control during emotional conflict monitoring. The early manifestation of anxiety in persons with Type 2 diabetes could be predicted and mediated by a patient’s glycaemic level and dACC–AMY functional connectivity. To our best knowledge, these findings provide the earliest neural evidence of emotional declines in diabetes before affective disorders are clinically diagnosable.

Along the same line, depressed patients also displayed hyperactivation of the dACC in an emotional Stroop study.^16^ According to the neural framework of emotionally salient stimuli influencing executive control, the ACC functions as the integrating hub that receives affective inputs from the AMY and then relays resources to the prefrontal cortex to carry out task goals.^42^ An increased but inefficient engagement of the dACC might imply that people with Type 2 diabetes perceived emotional conflicts to be more aversive than did control subjects and therefore required compensatory activity to downplay their affective significance. Still, this effortful engagement does not necessarily ensure improved task performance.^43^ As the dACC proved pivotal in assessing emotional salience amid the neurobiology of depression, this region is worth examining in further diabetes investigations.

Some limitation of this study must be acknowledged. First, the two groups of participants were not matched in gender. Nevertheless, the literature states that neither structural changes in the diabetes brain are not moderated by gender,^8^ and the emotional Stroop task has no gender disparities in clinical populations.^11,31^ Second, cerebrovascular comorbidities of Type 2 diabetes could disrupt neurovascular couplings and consequently attenuate blood oxygen level-dependent responses.^44^ While other vascular risk factors, such as smoking habit and alcohol consumption, were not assessed in this study, we used the cholesterol level, triglyceride level, blood pressure and BMI as proxies for controlling for the effect of the vascular risks, as the results of the complementary sensitivity tests. Worthy of note, the alcohol consumption was found to be not associated with any graph measures of functional connectivity index in Type 2 diabetes.^41^ Furthermore, the direct effect of BMI on cardiovascular outcomes was significant even after adjusting for the mediation effect of diabetes and smoking.^45^ Because BMI asserted a larger effect on the cardiovascular outcomes relative to diabetes and smoking, controlling for BMI in this study might render an underestimated diabetes effect of our dependent measures. Although the degree of vascular influence is less clear, we believed that the likelihood that our results were affected is low because (i) the change in blood oxygen level-dependent responses due to hyperglycaemia may be negligible;^46^ and (ii) instead of attenuated signals, we also observed certain regions with increased activities in diabetes relative to controls. Third, as cognition and emotion are interactively integrated,^42^ the inferior performance in people with Type 2 diabetes could not be solely attributed to cognitive or affective decrements. The inverse correlation between Δcon and the global cognitive ability (MoCA) might imply a cognitive factor. However, the lack of a direct link between psychological scales and experimental outcomes could reflect the fact that those scales might not be as sensitive as fMRI measures in preclinical stages. Along with recent perspectives suggesting that even completing a classic Stroop task could be understood as an emotional process since conflicts alone are aversive,^47^ our paradigm’s sensitivity to individuals with worsened emotional states is backed by plenty of emotional Stroop research.^11,31^

## Conclusions

‘It is preferable to incorporate psychosocial assessment and treatment into routine care’, pointed out in Standards of Medical Care in Diabetes.^5^ Diabetes-related affective declines may be relatively insidious compared to cognitive declines but must not be overlooked by healthcare providers. Physicians should continually monitor and assess the well-being of persons with diabetes since psychosocial interventions improved not only mental health outcomes but also HbA1c levels.^48^ Given that current recommended tools for such evaluations are predominantly self-reported scales,^49^ the emotional Stroop, a task that had been recognized in psychopathology, should have the potential to be added to those validated measures that can be applied in clinical settings.

## Supplementary Material

fcad022_Supplementary_Data

## Abbreviations

ACC =: anterior cingulate cortex
BAI =: Beck Anxiety Inventory
BDI-II =: Beck Depression Inventory (1996 revised edition)
BMI =: body mass index
CON =: congruent condition
dACC =: dorsal anterior cingulate cortex
DBP =: diastolic blood pressure
DDS =: Diabetes Distress Scale
DLPFC =: dorsolateral prefrontal cortex
DM =: diabetes mellitus
DST =: Digit Span Test
FPG =: fasting plasma glucose
HbA1c =: glycated haemoglobin
HOMA2-IR =: updated homoeostatic model assessment–insulin resistance
INCON =: incongruent condition
LDL-C =: low-density lipoproteins cholesterol
MoCA =: Montreal Cognitive Assessment
PPI =: psychophysiological interaction analysis
RTs =: reaction times
SBP =: systolic blood pressure
TG =: triglyceride
VLPFC =: ventrolateral prefrontal cortex
