# Supplementary material for: The neural correlates of emotional conflict monitoring as an early manifestation of affective and cognitive declines in persons with Type 2 diabetes
Source: Brain Commun. 2023 Feb 4;5(1):fcad022. doi: 10.1093/braincomms/fcad022 (PMC9945846; doi:10.1093/braincomms/fcad022)
Supplement: fcad022_Supplementary_Data [file fcad022_supplementary_data.pdf]

SUPPLEMENTARY MATERIALS

MRI Acquisition

Functional and structural MRI data were acquired on a 3T MRI scanner (Siemens Magnetom Tim Trio, Erlanger, Germany) equipped with a high-resolution 32-channel head array coil. For functional images, changes in BOLD T2*-weighted MR signals were collected along the AC–PC plane with a gradient echo-planar imaging (EPI) sequence (TR = 2200 ms, TE = 30 ms, field of view (FOV) = 220 mm, flip angle = 90°, matrix = 64 × 64, 36 transversal slices, voxel size = 3.4 × 3.4 × 3.0 mm^3, and no gap). High-resolution structural T1-weighted images were acquired using a 3D magnetization-prepared rapid gradient echo sequence (TR = 2530 ms, TE = 3.5 ms, FOV = 256 mm, flip angle = 7°, matrix = 256 × 256, 176 sagittal slices, voxel size = 1 × 1 × 1 mm^3, and no gap).

Image Preprocessing

Image processing and analysis were performed utilizing SPM12 (Wellcome Department of Imaging Neuroscience, London, UK; https://www.fil.ion.ucl.ac.uk/spm) in MATLAB 2020a (MathWorks, Sherborn, MA, USA). For details, please see Supplementary Materials. EPI images were reoriented, realigned and slice-time corrected. Structural scans were co-registered to the mean functional image, and a skull-stripped image was created from segmented gray matter, white matter, and cerebrospinal fluid (CSF) images, which were then combined to construct a subject-specific brain template. Co-registered EPI images were normalized to the standard Montreal Neurological Institute (MNI) space and smoothed with a 6-mm full width at half maximum (FWHM) Gaussian kernel.

Supplementary Table 1: Brain regions showing task activations of resolving emotional conflict (contrast incongruent (INCON)–congruent (CON)) in diabetes mellitus (DM) group and control group. All clusters were significant at a family-wise error (FWE)-corrected $p < 0.05$. Abbreviations: DM, diabetes mellitus; BA, Brodmann area; mPFC, medial prefrontal cortex; ACC, anterior cingulate cortex; PCC, posterior cingulate cortex.

INCON–CON	**Brain region**	BA	x	y	z	Peak T	Cluster size
Control	Inferior frontal gyrus	47	-38	20	-10	4.52	75
	mPFC	6	-16	-6	76	4.28	56
		6	12	-12	72	4.15	34
		6	-16	10	56	3.71	40
		10	20	40	-8	3.68	31
		6	20	-4	54	3.64	34
	Postcentral gyrus	3	-28	-18	52	3.91	52

	Precuneus	7	24	-62	46	3.09	36
	Anterior insula	13	-22	32	-2	3.41	57
	ACC	24	8	24	6	3.48	77
	PCC	30	-22	-42	20	4.27	111
	Hippocampus		38	-34	-4	3.34	23
	Parahippocampus	30	26	-44	14	4.65	332
		19	-34	-54	2	3.89	166
	Fusiform gyrus	19	-24	-72	0	3.82	80
	Thalamus		14	-24	20	3.63	24
	Midbrain		-8	-26	-12	4.59	166
			8	-26	-12	4.42	214
	Cerebellum		12	-48	-10	3.73	51
DM	Middle frontal gyrus	9	-54	22	34	4.79	1039
	Inferior frontal gyrus	46	-56	38	2	4.59	
	Anterior insula	13	-28	22	0	4.67	
	Middle frontal gyrus	8	54	16	34	4.70	516
	Inferior frontal gyrus	46	52	36	16	4.39	
	Inferior frontal gyrus	45	30	34	4	3.44	96
	mPFC	9	0	52	26	3.66	33
	ACC	24	-12	-2	26	3.77	104
		32	12	28	32	3.66	54
		32	-14	30	34	3.54	23
	PCC	31	14	-18	34	3.49	22
	Parahippocampus	30	-28	-50	8	3.24	23
	Thalamus		4	-10	0	3.23	26
	Midbrain		6	-26	-2	3.98	47

Supplementary Table 2: Group comparisons of fMRI results. (A) Brain regions showing task activations of resolving emotional conflict (contrast incongruent (INCON)–congruent (CON)). (B) Brain regions showing significant correlations between task activations of resolving emotional conflict (contrast INCON–CON) and the level of emotional interference (Δcon). All clusters were significant at a family-wise error (FEW)-corrected $p < 0.05$, except those marked with an asterisk, which were taken from predefined regions of interest (ROIs) and were significant at an uncorrected $p < 0.05$.

(A)	**Brain region**	BA	x	y	z	Peak T	Cluster size
Control > DM	VLPFC	47	-40	18	-10	3.76	83
	mPFC	10	-10	44	-14	3.62	79
	Parahippocampus	30	-12	-34	-6	3.30	49
	Hippocampus		-34	-36	-10	3.17	22
	Midbrain		8	-32	-14	3.87	57
	Cerebellum		10	-48	-14	3.96	58
DM > Control	dACC*	9	10	46	20	2.63*	78
	dACC*	32	12	28	32	2.60*	52
(B)	**Brain region**	BA	x	y	z	Peak T	Cluster size
Δcon as cov. (all groups)	dACC*	9	10	44	24	2.95*	12
	Anterior insula	13	-40	22	10	3.34	27

Abbreviations: DM, diabetes mellitus; BA, Brodmann area; VLPFC, ventrolateral prefrontal cortex; mPFC, medial prefrontal cortex; dACC, dorsal anterior cingulate cortex; cov., covariate.

Supplementary Figure 1: Brain regions showing task activations of resolving emotional conflict (contrast INCON – CON) in DM (cyan) and Control group (orange).

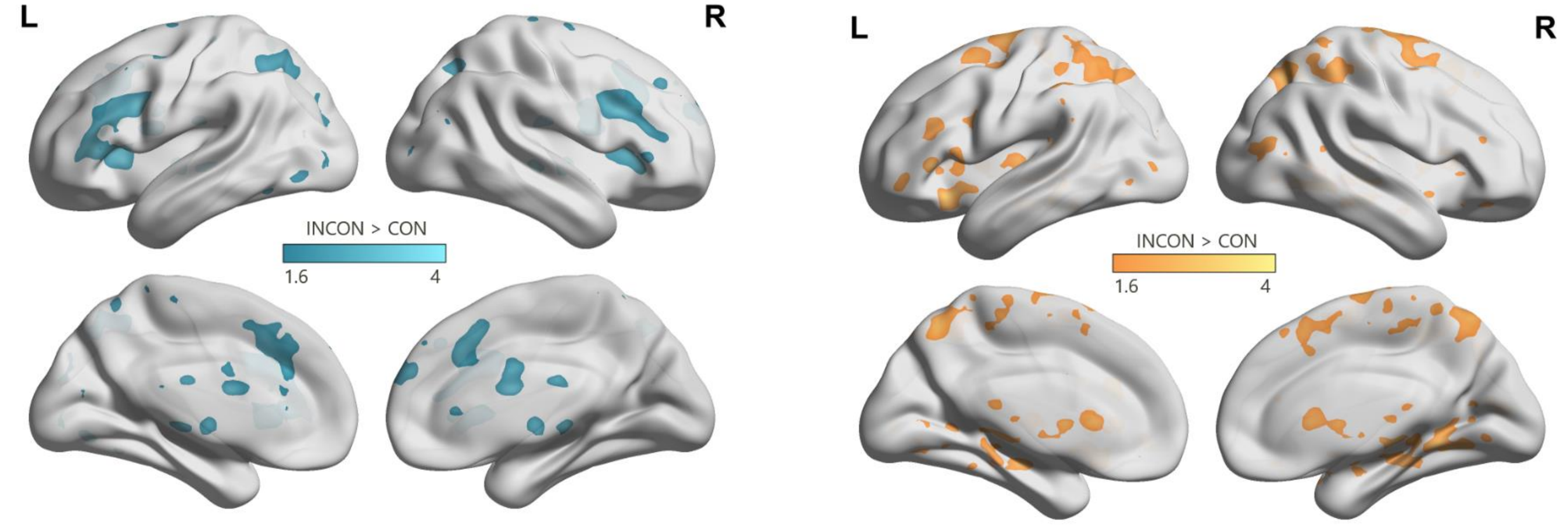

Supplementary Figure 2: Brain regions showing task activations of resolving emotional conflict (contrast INCON – CON) between groups (orange: Control group > DM group; cyan: DM group > Control group). Regions that survived the thresholds at FWE-corrected $p < 0.05$ were labeled.

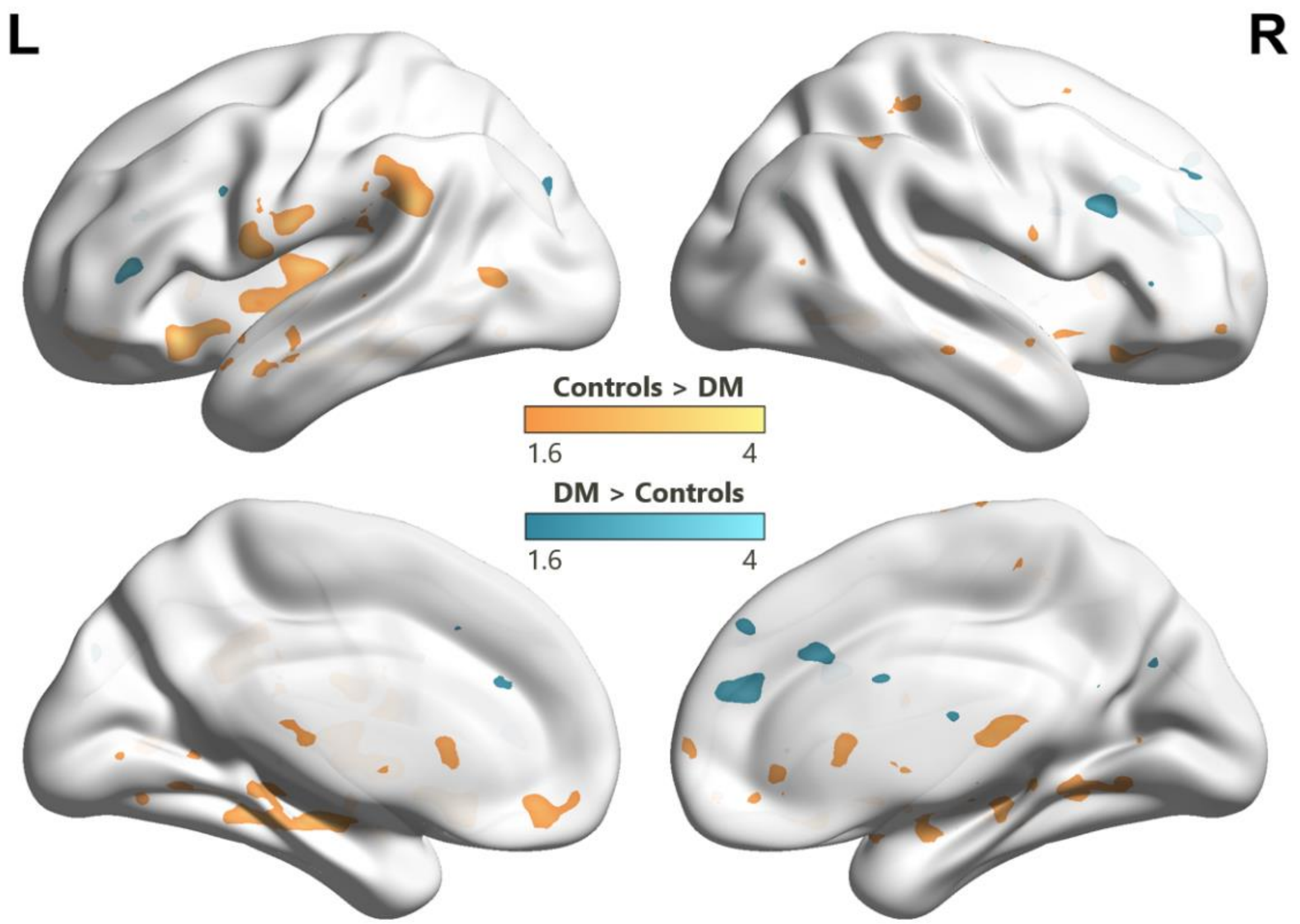

Supplementary Figure 3: dACC–DLPFC functional connectivity (M) mediates the relationship between Δcon (X) and MoCA (Y). (A) Path diagram demonstrates the relationship between variables in the path model. Δcon (left) as the predictor variable predicts the MoCA (right). The connection of Δcon to the mediator (dACC–DLPFC functional connectivity) as mediator is the a path. The lines are labeled with path coefficients, and standard errors are shown in parentheses. The connection of the mediator (dACC–DLPFC functional connectivity) to the outcome (MoCA) is the b path. They are calculated controlling for Δcon, as the standard in mediation models. *** $p < 0.001$, ** $p < 0.01$, * $p < 0.05$, two-tailed. The direct path is the **c'** path, which is calculated controlling for brain mediator. (B) Substantiation of the mediation path **a**, **b** and **c**. Regression scatterplots depict the relationships between predictor (i.e., Δcon) and dACC–DLPFC connectivity (path **a**). Partial regression scatterplots demonstrate the relationships between dACC–DLPFC connectivity and MoCA (path **b**). (C) The mediation effect (**a***b**) is substantiated by the bootstrapped distributions. The range on the x-axis spanned by the lighter gray portion of the histogram is the 95% confidence interval for the effect.

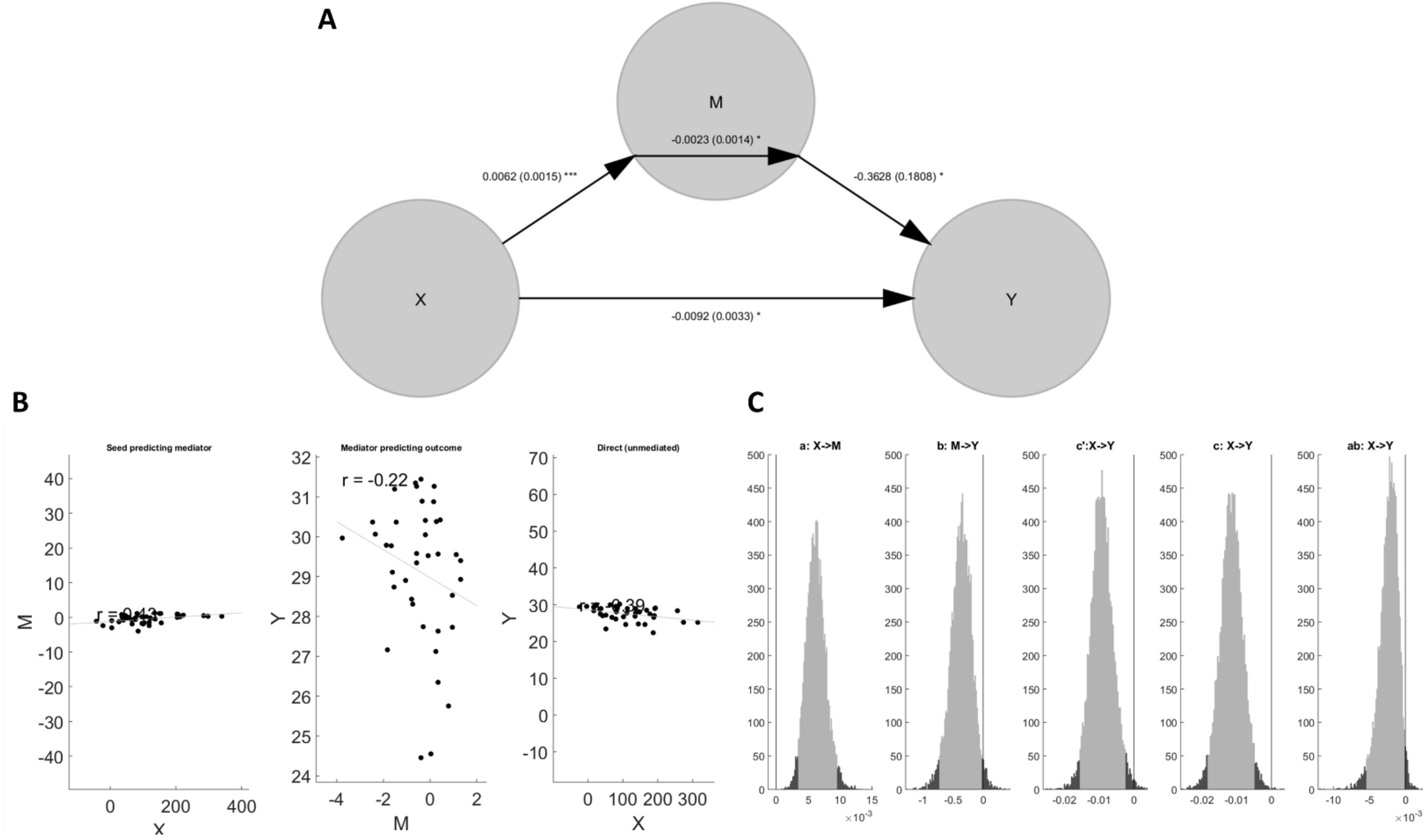
A
M
X
Y
0.0062 (0.0015) ***
-0.0023 (0.0014) *
-0.3628 (0.1808) *
-0.0092 (0.0033) *
B
Seed predicting mediator
Mediator predicting outcome
Direct (unmediated)
r = -0.22
M
X
Y
C
a: X->M
b: M->Y
c':X->Y
c: X->Y
ab: X->Y
×10⁻³

Supplementary Figure 4: dACC–VLPFC functional connectivity (M) mediates the relationship between Δcon (X) and MoCA (Y). (A) Path diagram demonstrates the relationship between variables in the path model. Δcon (left) as the predictor variable predicts the MoCA (right). The connection of Δcon to the mediator (dACC–VLPFC functional connectivity) as mediator is the a path. The lines are labeled with path coefficients, and standard errors are shown in parentheses. The connection of the mediator (dACC–VLPFC functional connectivity) to the outcome (MoCA) is the b path. They are calculated controlling for Δcon, as the standard in mediation models. *** $p < 0.001$, ** $p < 0.01$, * $p < 0.05$, two-tailed. The direct path is the c' path, which is calculated controlling for brain mediator. (B) Substantiation of the mediation path a, b and c. Regression scatterplots depict the relationships between predictor (i.e., Δcon) and dACC–VLPFC connectivity (path a). Partial regression scatterplots demonstrate the relationships between dACC–VLPFC connectivity and MoCA (path b). (C) The mediation effect (**a***b**) is substantiated by the bootstrapped distributions. The range on the x-axis spanned by the lighter gray portion of the histogram is the 95% confidence interval for the effect.

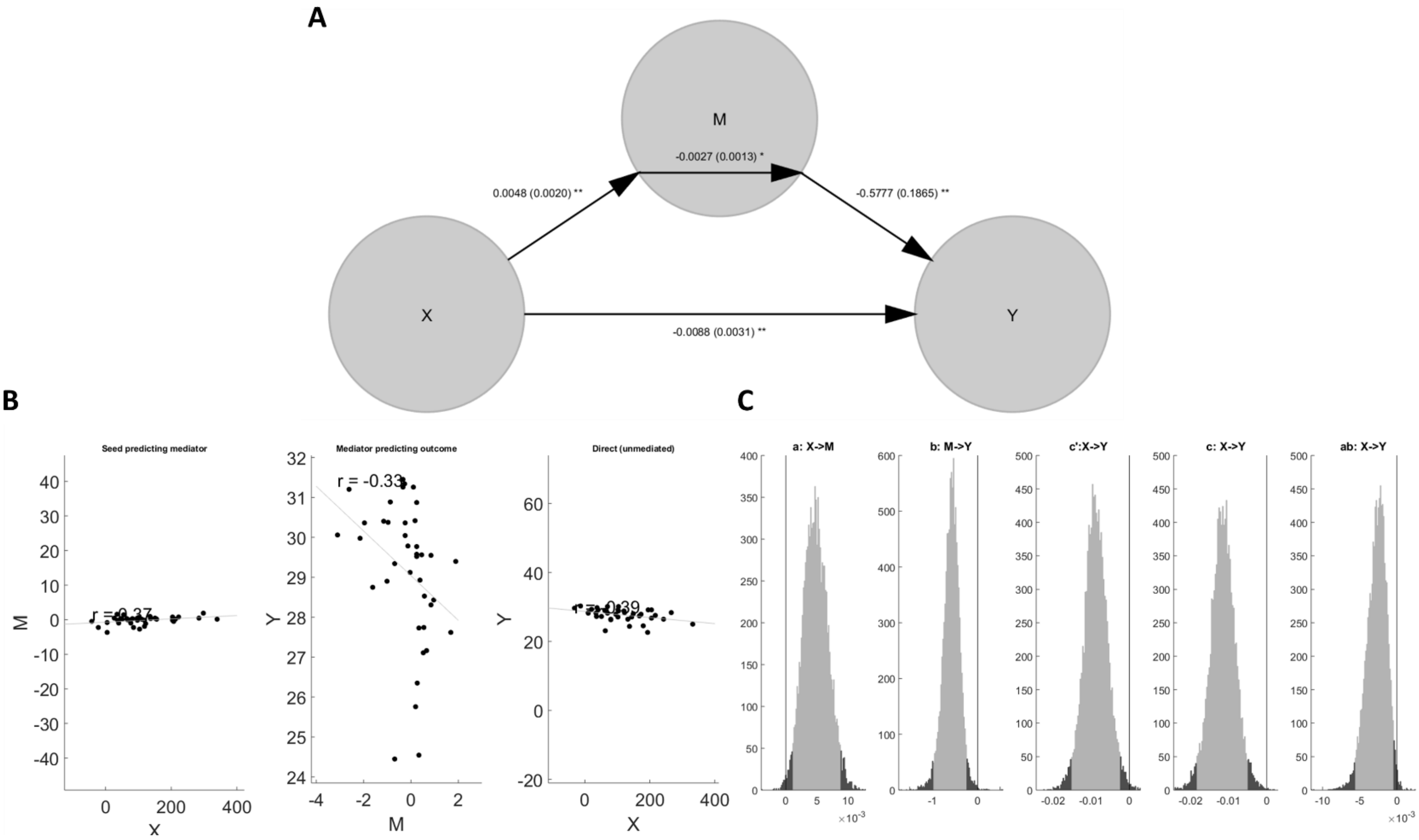

A
M
X
Y
-0.0027 (0.0013) *
0.0048 (0.0020) **
-0.5777 (0.1865) **
-0.0088 (0.0031) **
B
C
Seed predicting mediator
Mediator predicting outcome
Direct (unmediated)
r = -0.33
a: X->M
b: M->Y
c':X->Y
c: X->Y
ab: X->Y

Supplementary Figure 5: dACC–amygdala functional connectivity (M) mediates the relationship between HbA1c (X) and Beck Anxiety score (Y). (A) Path diagram demonstrates the relationship between variables in the path model. HbA1c (left) as the predictor variable predicts the Beck Anxiety score (right). The connection of HbA1c to the mediator (dACC–amygdala functional connectivity) as mediator is the a path. The lines are labeled with path coefficients, and standard errors are shown in parentheses. The connection of the mediator (dACC–amygdala functional connectivity) to the outcome (BAI) is the b path. They are calculated controlling for HbA1c, as the standard in mediation models. *** $p < 0.001$, ** $p < 0.01$, * $p < 0.05$, two-tailed. The direct path is the **c'** path, which is calculated controlling for brain mediator. (B) Substantiation of the mediation path **a**, **b** and **c**. Regression scatterplots depict the relationships between predictor (i.e., HbA1c) and dACC–amygdala connectivity (path **a**). Partial regression scatterplots demonstrate the relationships between dACC–amygdala connectivity and BAI (path **b**). (C) The mediation effect (**a***b**) is substantiated by the bootstrapped distributions. The range on the x-axis spanned by the lighter gray portion of the histogram is the 95% confidence interval for the effect.

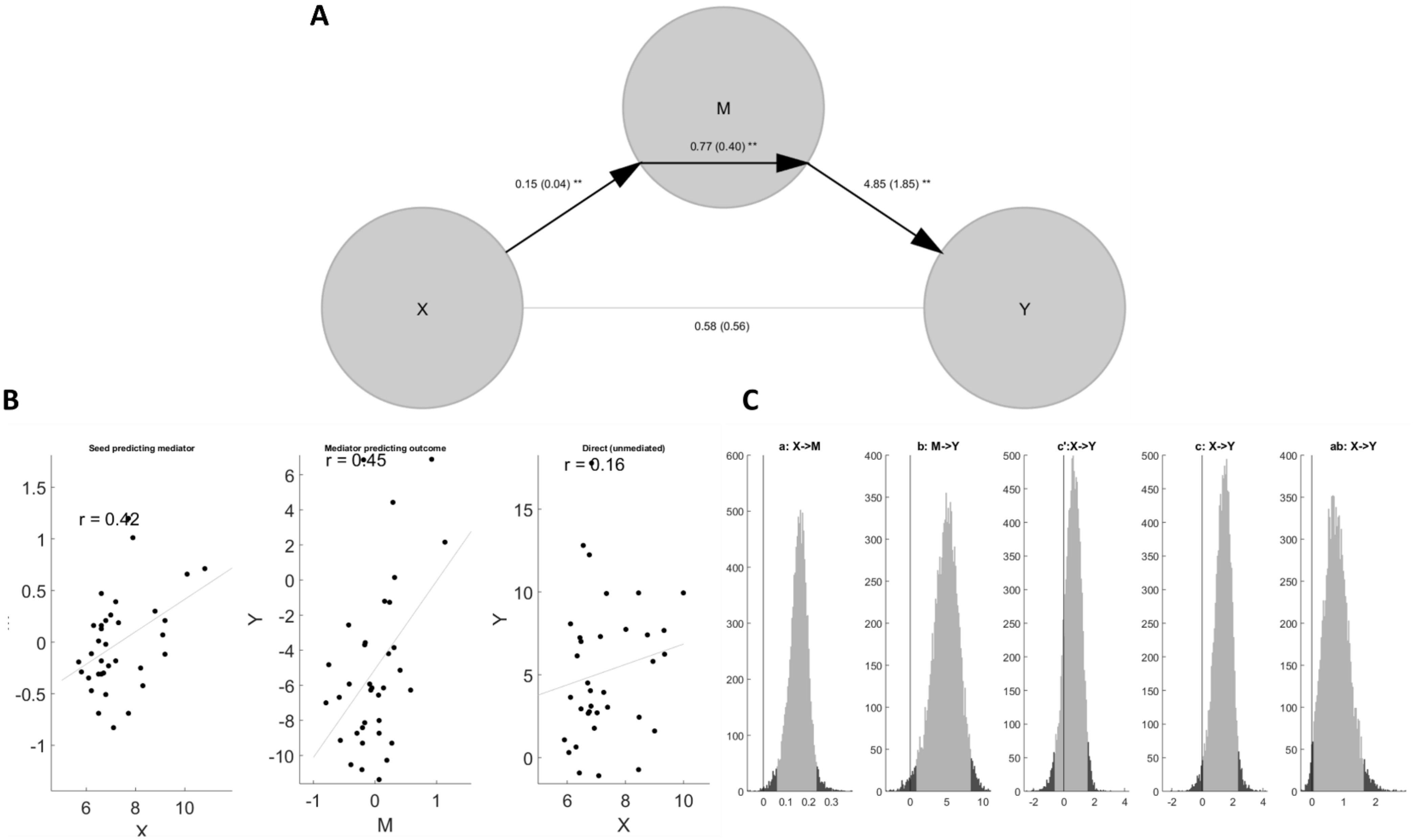
A
M
X
Y
0.77 (0.40) **
0.15 (0.04) **
4.85 (1.85) **
0.58 (0.56)
B
Seed predicting mediator
r = 0.42
Mediator predicting outcome
r = 0.45
Direct (unmediated)
r = 0.16
C
a: X->M
b: M->Y
c':X->Y
c: X->Y
ab: X->Y

Generated codes

```
clear all;
close all;

%----------%

%---Block -----%
BlockNumber=12;
%---------------%

%---Trial -----%
TrialNumber=8;
%---------------%

%-------------------%
% example:  1=blue 2=red 3=yellow
%         ColorIndex=[1 2 3];
%         1=blue 2=red
%         ColorIndex=[1 2];
ColorIndex=[1 2 3];

% color matrix %

color2=repmat(ColorIndex, 1, TrialNumber*3/length(ColorIndex)); % for conscious, angry
color3=repmat(ColorIndex, 1, TrialNumber*3/length(ColorIndex)); %
color5=repmat(ColorIndex, 1, TrialNumber*3/length(ColorIndex));
```

```
color6=repmat(ColorIndex, 1, TrialNumber*3/length(ColorIndex));

%-------------------------------%

% ---- ------------%
% initialize Key
leftKey=KbName('2@');   %
middleKey=KbName('4$'); %
rightKey=KbName('6^');  %
%--------------------------------%

%-----------%
%
% Example:
% Datatype=2
DataType=2;
%-------------------------%

%--------%
%
%
f=[5 6 11 3 10 1 4 9 2 7 8 1 6 5 4 11 3 9 10 4 8 2 5 7 ];
n=[5 6 11 3 10 1 4 9 2 7 8 1 6 5 4 11 3 9 10 4 8 2 5 7 ];
%
mf1=[6  1  3  8  5  10  9  2  7  4  1  11  5  9  8  3  6  2  1  11  4  10  7  3;
    7  2  4  9  1  11  6  5  10  2  3  8  4  7  6  5  10  3  5  9  1  8  11  4];
mn1=[6  1  3  8  5  10  9  2  7  4  1  11  5  9  8  3  6  2  1  11  4  10  7  3;
```

```
    7  2  4  9  1  11  6  5  10  2  3  8  4  7  6  5  10  3  5  9  1  8  11  4];

save('Fear1', 'f');
save('Neutral1', 'n');
save('MaskedFear1', 'mf1');
save('MaskedNeutral1', 'mn1');

%------------------------%

%---- --%
%load Condition1;
condition=[6 5 3 2 6 3 5 5 6 2 3 2]; %
           % 2=ConsciousFear 3=ConsciousNeutral 5=NonconsciousFear
           % 6=NonconsciousNeutral
           % Example [2 3 5 6]
save('Condition', 'condition');
%------------------------%

%----------%
textfontSize=24;
textColor1=[128 128 128];
textColor2=[255 255 255];
textColor3=[0 0 0];
text='+';
% ----------------------%

%-----------------------------------------------------------------------%
```

```
% Initialize record matrix
Response2=zeros(DataType,TrialNumber*BlockNumber);% column1 response column2

%--Initialize index----%
countAll=1;
%countCA=1;
countCF=1; countCN=1;
%countUA=1;
countUF=1; countUN=1;
%---------------------%



% generate texture index
%CAtex=zeros(1,12);
CFtex=zeros(1,TrialNumber*BlockNumber);
CNtex=zeros(1,TrialNumber*BlockNumber);

%UAtex=zeros(2,12); % Row1 for target Row2 for mask
UFtex=zeros(2,TrialNumber*BlockNumber);
UNtex=zeros(2,TrialNumber*BlockNumber);

%-------------------%
% Open a window
bkgColor=[0 0 0];
whichScreen=0;
[theWindow, theRect]=Screen('OpenWindow',whichScreen, bkgColor, []);
```

```
HideCursor;
% Setting center
centerX=theRect(3)/2;
centerY=theRect(4)/2;
%----------------------------%

%--------------------CF------------------------------------------%
% Random Color
index=randperm(length(color2));
for i=1:length(color2)
 temp=color2(index(i));
 color2(index(i))=color2(i);
 color2(i)=temp;
end
save('ColorMatrix2', 'color2');
% for CFtex
for i=1:length(color2)
    c=color2(i); % 1=blue 2=red 3=yellow
    switch c
        case{1} %blue
          order=num2str(f(i));
          img=imread([order 'fb'], 'bmp');
          CFtex(i)=Screen('MakeTexture', theWindow, img,[],1);
        case{2} %red
          order=num2str(f(i));
          img=imread([order 'fr'], 'bmp');
          CFtex(i)=Screen('MakeTexture', theWindow, img,[],1);
```

```
        case{3} %yellow
          order=num2str(f(i));
          img=imread([order 'fy'], 'bmp');
          CFtex(i)=Screen('MakeTexture', theWindow, img,[],1);
    end
end
%---------------------------------------------------------------%

%-------------------------CN------------------------------------%
% Random Color
index=randperm(length(color3));
for i=1:length(color3)
 temp=color3(index(i));
 color3(index(i))=color3(i);
 color3(i)=temp;
end
save('ColorMatrix3', 'color3');
% for CNtex
for i=1:length(color3)
    c=color3(i); % 1=blue 2=red 3=yellow
    switch c
        case{1} %blue
          order=num2str(n(i));
          img=imread([order 'nb'], 'bmp');
          CNtex(i)=Screen('MakeTexture', theWindow, img,[],1);
        case{2} %red
          order=num2str(n(i));
```

```
        img=imread([order 'nr'], 'bmp');
        CNtex(i)=Screen('MakeTexture', theWindow, img,[],1);
       case{3}
        order=num2str(n(i));
        img=imread([order 'ny'], 'bmp');
        CNtex(i)=Screen('MakeTexture', theWindow, img,[],1);
    end
end

%---------------------UF-----------------------------------%
% Random Color
index=randperm(length(color5));
for i=1:length(color5)
 temp=color5(index(i));
 color5(index(i))=color5(i);
 color5(i)=temp;
end
save('ColorMatrix5', 'color5');

% for UFtex
for i=1:length(color5)
    c=color5(i); % 1=blue 2=red 3=yellow
    switch c
       case{1} %blue
        order=num2str(mf1(1,i));
        order2=num2str(mf1(2,i));
        img=imread([order 'fb'], 'bmp');
```

```
        img2=imread([order2 'nb'], 'bmp');
        UFtex(1,i)=Screen('MakeTexture', theWindow, img,[],1);
        UFtex(2,i)=Screen('MakeTexture', theWindow, img2,[],1);
      case{2} %red
        order=num2str(mf1(1,i));
        order2=num2str(mf1(2,i));
        img=imread([order 'fr'], 'bmp');
        img2=imread([order2 'nr'], 'bmp');
        UFtex(1,i)=Screen('MakeTexture', theWindow, img,[],1);
        UFtex(2,i)=Screen('MakeTexture', theWindow, img2,[],1);
      case{3}
        order=num2str(mf1(1,i));
        order2=num2str(mf1(2,i));
        img=imread([order 'fy'], 'bmp');
        img2=imread([order2 'ny'], 'bmp');
        UFtex(1,i)=Screen('MakeTexture', theWindow, img,[],1);
        UFtex(2,i)=Screen('MakeTexture', theWindow, img2,[],1);
    end
end

%------------------------UN----------------------------------%
% Random Color
index=randperm(length(color6));
for i=1:length(color6)
 temp=color6(index(i));
 color6(index(i))=color6(i);
 color6(i)=temp;
```

```
end
save('ColorMatrix6', 'color6');
% for UNtex
for i=1:length(color6)
    c=color6(i); % 1=blue 2=red 3=yellow
    switch c
        case{1} %blue
          order=num2str(mn1(1,i));
          order2=num2str(mn1(2,i));
          img=imread([order 'nb'], 'bmp');
          img2=imread([order2 'nb'], 'bmp');
          UNtex(1,i)=Screen('MakeTexture', theWindow, img,[],1);
          UNtex(2,i)=Screen('MakeTexture', theWindow, img2,[],1);
        case{2} %red
          order=num2str(mn1(1,i));
          order2=num2str(mn1(2,i));
          img=imread([order 'nr'], 'bmp');
          img2=imread([order2 'nr'], 'bmp');
          UNtex(1,i)=Screen('MakeTexture', theWindow, img,[],1);
          UNtex(2,i)=Screen('MakeTexture', theWindow, img2,[],1);
        case{3}
          order=num2str(mn1(1,i));
          order2=num2str(mn1(2,i));
          img=imread([order 'ny'], 'bmp');
          img2=imread([order2 'ny'], 'bmp');
          UNtex(1,i)=Screen('MakeTexture', theWindow, img,[],1);
          UNtex(2,i)=Screen('MakeTexture', theWindow, img2,[],1);
```

```
    end
end

clear temp color order order2 img img2;

%-------------------------- Wiat for trigger ----------------------------%
dio=digitalio('parallel','LPT1');
hwlines=addline(dio,0:4,1,'in');
temp=binvec2dec(getvalue(dio));
signal=0;

img=imread('instruction', 'bmp');
LookAt=Screen('MakeTexture', theWindow, img);
Screen('DrawTexture', theWindow, LookAt);
Screen('Flip', theWindow);

while signal==0
   trigger=getvalue(dio);
   trigger=binvec2dec(trigger);
    if  temp~=trigger
        signal=1;
   end
end
signal=0;
%-------------------------- Wiat for trigger ----------------------------%

%-------------------------Display
```

```
%face-----------------------------------%

for block=1:BlockNumber
   c=condition(block);

   % Resting State for 13.2s
     Screen('TextSize', theWindow, textfontSize);
     Screen('DrawText', theWindow, text, centerX-12, centerY-12, textColor1);
     Screen('Flip', theWindow);
     WaitSecs(17.60);

     % ----------Wait for trigger -----------%
     while signal==0
        trigger=getvalue(dio);
        trigger=binvec2dec(trigger);
         if  temp~=trigger
             signal=1;
         end
      end
      signal=0;
     % ----------Start-----------------------%

     for trial=1:TrialNumber

       % Draw a + fixation for 200ms
       Screen('TextSize', theWindow,  textfontSize);
       Screen('DrawText', theWindow, text, centerX-12, centerY-12, textColor2);
```

```
Screen('Flip', theWindow);
WaitSecs(0.2);

% Blank for 400ms
Screen('DrawText', theWindow, text, centerX, centerY, textColor3);
Screen('Flip', theWindow);
WaitSecs(0.4);

 % Show  face
 switch c
       %case{1} % angry
       %    Screen('DrawTexture', theWindow, CAtex(countCA), [], destRect, 0);
       %    Screen('Flip', theWindow, [], [], 2);
       %    WaitSecs(0.2);
       %    Screen('Close', CAtex(countCA));
       %    countCA=countCA+1;
       case{2} % fear
           Screen('DrawTexture', theWindow, CFtex(countCF), [], [], 0);
           Screen('Flip', theWindow, [], [], 2);
           WaitSecs(0.2);
           Screen('Close', CFtex(countCF));
           countCF=countCF+1;
       case{3} % neutral
           Screen('DrawTexture', theWindow, CNtex(countCN), [], [], 0);
           Screen('Flip', theWindow, [], [], 2);
           WaitSecs(0.2);
           Screen('Close', CNtex(countCN));
```

```
    countCN=countCN+1;

% case{4} % unconsious angry
%     Screen('DrawTexture', theWindow, UAtex(1,countUA), [], destRect, 0);
%     Screen('Flip', theWindow, [], [], 2);
%     WaitSecs(0.2);

%     Screen('DrawTexture', theWindow, UAtex(2, countUA), [], destRect, 0);
%     Screen('Flip', theWindow, [], [], 2);
%     WaitSecs(0.2);
%     Screen('Close', [UAtex(1,countUA), UAtex(2,countUA)]);
%     countUA=countUA+1;

 case{5} % unconsious fear
    Screen('DrawTexture', theWindow, UFtex(1,countUF), [], [], 0);
    Screen('Flip', theWindow, [], [], 2);
    WaitSecs(0.017);
    Screen('DrawTexture', theWindow, UFtex(2, countUF), [], [], 0);
    Screen('Flip', theWindow, [], [], 2);
    WaitSecs(0.183);
    Screen('Close', [UFtex(1,countUF), UFtex(2,countUF)]);
    countUF=countUF+1;

 case{6} % unconsious neutral
    Screen('DrawTexture', theWindow, UNtex(1,countUN), [], [], 0);
    Screen('Flip', theWindow, [], [], 2);
    WaitSecs(0.017);
```

```
                Screen('DrawTexture', theWindow, UNtex(2, countUN), [], [], 0);
                Screen('Flip', theWindow, [], [], 2);
                WaitSecs(0.183);
                Screen('Close', [UNtex(1,countUN), UNtex(2,countUN)]);
                countUN=countUN+1;
        end
        % Blank for response
          Screen('DrawText', theWindow, text, centerX, centerY, textColor3);
          Screen('Flip', theWindow);
          [Response2(1,countAll), Response2(2,countAll)]=my_waitForButtonPress2(1.33, leftKey, middleKey, rightKey);
          countAll=countAll+1;
    end
end

%§ó§ïÀÉ¦W
save('ASD01_run1', 'Response2');
Screen('CloseAll');
ShowCursor;
```
